# Lessons learned from a sporadic FUSopathy in a young man: a case report

**DOI:** 10.1186/s12883-023-03082-0

**Published:** 2023-02-02

**Authors:** Ernesto García-Roldán, Eloy Rivas-Infante, Manuel Medina-Rodríguez, José Enrique Arriola-Infante, Silvia Rodrigo-Herrero, Carmen Paradas, Alberto Rábano-Gutiérrez, Emilio Franco-Macías

**Affiliations:** 1 Department of Neurology, Memory Unit, Institute of Biomedicine of Seville, Hospital Universitario Virgen del Rocío, Sevilla, Spain; 2 Anatomic Pathology Department, Hospital Universitario Virgen del Rocío, Sevilla, Spain; 3 Department of Neurology, Neurovascular Unit, Institute of Biomedicine of Seville, Hospital Universitario Virgen del Rocío, Sevilla, Spain; 4 Department of Neurology, Memory Unit, Hospital Universitario Virgen del Rocío, Sevilla, Spain; 5 Department of Neurology, Memory Unit, Hospital Universitario Juan Ramón Jiménez, Huelva, Spain; 6 Department of Neurology, Neuromuscular Disorders Unit, Institute of Biomedicine of Seville, Hospital Universitario Virgen del Rocío/CSIC, Universidad de Sevilla, CIBERNED, Sevilla, Spain; 7 Fundación CIEN, Madrid, Spain; 8 Department of Neurology, Memory Unit, Hospital Universitario Virgen del Rocío/Centro de Neurología Avanzada (CNA), Sevilla, Spain

**Keywords:** Neurodegenerative diseases, Frontotemporal lobar degeneration, RNA-Binding protein FUS

## Abstract

**Background:**

In frontotemporal dementia (FTD) spectrum, younger patients may correspond to fusopathy cases, and cognitive decline could be rapidly progressive. We present a clinical and neuropathological description of a patient.

**Case presentation:**

A 37-year-old man, without a family history of neurodegenerative diseases, was brought by his family to consult for dysarthria and behavioural change. Initial exploration showed spastic dysarthria and disinhibition. He progressively worsened with a pseudobulbar syndrome, right-lateralized pyramidal signs, left hemispheric corticobasal syndrome and, finally, lower motor neuron signs in his right arm. He died four years after the initiation of the syndrome from bronchopneumonia. Laboratory tests (including blood and cerebrospinal fluid (CSF)) were normal. Magnetic resonance imaging (MRI) and fluorodeoxyglucose-containing positron emission tomography (PET-^18^F-FDG) showed left fronto-insular atrophy and hypometabolism. Subsequently, 123I-ioflupane (DaT-SCAN®) single-photon emission computed tomography (SPECT) was pathologic, manifesting bilaterally decreased activity with greater affection on the left side. Only a third electromyogram (EMG) detected denervation in the last year of evolution. No mutations were found in genes such as Tau, progranulin, C9orf72, FUS, TDP-43, CHMP2B, or VCP. In necropsy, severe frontotemporal atrophy with basophilic neuronal cytoplasmic and intranuclear inclusions, negative for tau and TAR DNA binding protein 43 (TDP-43), but positive for fused in sarcoma (FUS) consistent with specifically basophilic inclusions body disease (BIBD) type was found.

**Conclusions:**

In patients affected by FTD, particularly the youngest, with rapidly progressive decline and early motor affection, fusopathy must be suspected. These cases can include motor signs described in the FTD spectrum. Lower motor neuron affection in EMG could be detected late.

## Background

FTD is a heterogeneous neurodegenerative disease associated with frontal and temporal atrophy. A wide range of signs and symptoms are associated, from cognitive dysfunction to neurological signs of movement disorders spectrum and motor neuron syndrome. Fusopathy cases may correspond to younger patients who suffer rapid cognitive decline.

## Case presentation

A 37-year-old right-handed man presented progressive speaking difficulty for the previous six months. The family history was negative for any neurodegenerative disease. When first examined at age 37 his speech was effortful, hesitating at the beginning of each sentence and speaking syllable by syllable in a telegraphic way lacking in abstraction. Phonological errors in naming tasks, decreased fluency by letters, and difficulties in Similarities tests were also detected. In addition, his family had noticed behavioural changes in the patient during the previous year. His behaviour seemed childish with some disinhibition and loss of manners. Dietary changes with a preference for sweets and, subsequently, weight gain were also reported. At that time, an MRI showed left fronto-insular atrophy, and PET-^18^F-FDG revealed hypometabolism in the same region. Blood and CSF examinations were not remarkable. A first electromyogram, including bulbar muscles, was normal. No mutation was identified by whole exome sequencing test (WES) in the genes for tau, progranulin, C9ORF72, FUS, TDP-43, CHMP2B, or VCP.

Throughout the following year, the changes in the behaviour of the patient were getting worse with a loss of decorum, empathy, and compulsive actions. Dysarthria worsened, and then dysphagia emerged. At 38 years old, marked utilization behaviour and lack of insight were already evident. The neurological examination also revealed spastic dysarthria, difficulty mobilizing the soft palate, and increased jaw and gag reflexes, all consistent with the pseudobulbar syndrome. Deep tendon reflexes were slightly increased in all limbs, with right predominance. The patient did not show tongue atrophy or fasciculations either in the initial or middle phase of the illness, and no other muscle weakness, atrophy, or fasciculation was found. A second electromyogram was again within normal limits.

At 39 years of age, on examination, his gait was already impaired, mutism was already evident, and oculomotor and buccofacial apraxia had emerged. Right rigidity, bradykinesia, and ideomotor apraxia were first observed and, at that time, a DaT-SCAN® showed bilateral low dopamine transporter uptake (the specific binding ratio from the right striatum was 26,596 and from the left striatum was 22,483). Six months later, paresis and atrophy were first observed in his right forearm and hand on a background of upper motor neuron signs with a right predominance. He became bedridden at 39 years old. At that time, a third electromyogram showed diffuse denervation, and a computer tomography scan showed generalized frontal and temporal atrophy. He died from bronchopneumonia at 41 years of age. His disease duration was almost four years.

**Fig. 1:**
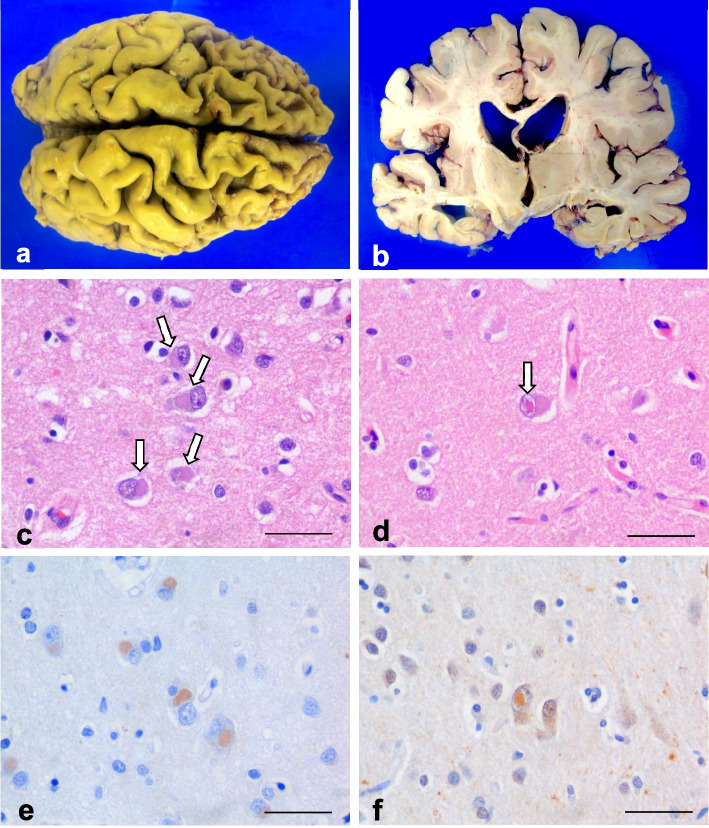
Neuropathological features of FTLD-ALS with FUS positive inclusions. Post-mortem examination confirmed a severe fronto-temporal atrophy and caudate nucleus degeneration (**a**-**b**) Images corresponds to the left hemisphere (the most affected). Histological examination revealed numerous compact basophilic cytoplasmic inclusions in cortical neurons (H&E, **c**) that are positive with FUS immunohistochemistry (**e**). Some ubiquitin-positive neuronal intranuclear inclusions (NIIs) were identified in cortex (H&E, **d** and ubiquitin IHC, **f**). Scale Bars = 50 μm

A post-mortem examination confirmed a reduced brain weight (1138 g) and a marked asymmetric frontotemporal lobar degeneration, with severe caudate nucleus atrophy and moderate pallidus nucleus involvement. Histological examination with hematoxylin and eosin (H&E) revealed cortical neuronal loss and reactive gliosis and identified abundant basophilic neuronal cytoplasmic inclusions (NCIs). Specifically, moderate pyramidal neuron loss and reactive gliosis were found in the primary motor cortex. Besides this, moderate myelin pallor was evident in the pyramidal tract and mild signs of degeneration were present in some motor neurons of the lower motor neuron pool. The substantia nigra showed mild loss of pigmented neurons, macrophages with neuromelanin and reactive astrogliosis, but no Lewy bodies and basophilic inclusions were identified. FUS immunohistochemistry highlighted much more widespread NCIs with a wide spectrum of morphologies in the neocortex, hippocampus, and subcortical regions. Few FUS negative neuronal intranuclear inclusions (NIIs) were also recognized with H&E and ubiquitine. Basophilic NCIs, characteristic of the basophilic inclusion body disease (BIBD) subtype of fusopathy, were found (Fig. 1). No beta-amyloid or alpha-synuclein were identified, and only very occasional TAU-positive neurofibrillary tangles in the amygdala and neuritic plaques in the hippocampus were found.

## Discussion and conclusions

The final diagnosis was frontotemporal lobar degeneration with amyotrophic lateral sclerosis (FTLD-ALS) with FUS-positive inclusions. FTLD cases are now assigned to one of the three major molecular subgroups based on histopathological findings: frontotemporal lobar degeneration with microtubule-associated tau (FTLD-tau), frontotemporal lobar degeneration with TAR DNA binding protein 43 inclusion bodies (FTLD-TDP-43), or FTLD-FUS [1]. FTLD-FUS is the least common of them and occurs in approximately 5% of patients, but the youngest patients of the entire spectrum belong to this subgroup. Usually, these cases are sporadic, and three subtypes are described: atypical FTLD-U (aFTLD-U), basophilic inclusion body disease (BIBD), and neuronal intermediate filament inclusion disease (NIFID) [2]. The neuropathological examination of this patient was consistent with the BIBD subtype (Fig. 1).

The largest reported series of BIBD included a total of 8 cases with an average age of onset of 46 years (ranging from 29 to 57). The average duration of the disease was 8 years. BIBD cases usually present with behavioural symptoms. Most of the patients subsequently develop motor neuron disease. Severe dysarthria has also been described at the time of onset, in addition to behavioural symptoms [3]. The onset of pseudobulbar syndrome in this patient could determine a shorter survival. The emergence of a corticobasal syndrome made us consider the FTLD-tau subtype, but atypical parkinsonism, associated with basal ganglia involvement, has also been described in a few cases of BIBD [4]. The BIBD subtype has a rapidly progressive course which could include all the clinical spectrum of FTD from atypical parkinsonism to motor neuron disease [3]. Neuropathology post-mortem examination remains essential for final diagnosis, and a complete immunohistochemical panel is crucial to identify FUS-positive inclusions and confirm the specific FTLD subtype. Regarding the newest trends in the knowledge of the role of FUS [5], we think that it would be recommended the study of the functions related to nuclear and cytoplasmatic alterations. It is essential to understand how FUS interacts with DNA and RNA because these proteins have a wide range of major functions involving these cardinal molecules. It is involved in various steps of the metabolism of DNA and RNA, so it might not be as good as therapeutical target because we can harm cells easily if we use gain-of-function or loss-of-function drugs. However, if we know how to change these functions indirectly through their modifying factors or cellular conditions, we could also know new potential therapeutical targets.

## Data Availability

The datasets used and analysed during the current study are available from the corresponding author on reasonable request.

## Abbreviations

BIBD: Basophilic inclusions body disease
CSF: Cerebrospinal fluid
DaT-SCAN®: 123I-ioflupane
DNA: Deoxyribonucleic acid
EMG: Electromyogram
FTD: Frontotemporal dementia
aFTLD-U: Atypical FTLD
FTLD: Frontotemporal lobar degeneration
FTLD-ALS: Frontotemporal lobar degeneration with amyotrophic lateral sclerosis
FTLD-FUS: Frontotemporal lobar degeneration with fused in sarcoma positive inclusions
FTLD-Tau: Frontotemporal lobar degeneration with microtubule-associated tau
FTLD-TDP-43: Frontotemporal lobar degeneration with TAR DNA binding protein 43 inclusion bodies
FUS: Fused in sarcoma
H&E: Hematoxylin and eosin
MRI: Magnetic resonance imaging
NCI: Neuronal cytoplasmic inclusions
NIFID: Neuronal intermediate filament inclusion disease
NII: Neuronal intranuclear inclusions
PET-^18^F-FDG: Fluorodeoxyglucose-containing positron emission tomography
RNA: Ribonucleic acid
SPECT: Single-photon emission computed tomography
WES: Whole Exome Sequencing test
